# Study protocol for Attachment & Child Health (ATTACH^TM^) program: promoting vulnerable Children’s health at scale

**DOI:** 10.1186/s12887-022-03439-3

**Published:** 2022-08-19

**Authors:** Lubna Anis, Nicole Letourneau, Kharah M. Ross, Martha Hart, Ian Graham, Simone Lalonde, Suzanna Varro, Alanna Baldwin, Angela Soulsby, Annette Majnemer, Carlene Donnelly, Caroline Piotrowski, Carrie Collier, Cliff Lindeman, Dan Goldowitz, Dawn Isaac, Denise Thomson, Diane Serré, Elisabeth Citro, Gabrielle Zimmermann, Harold Pliszka, Jackie Mann, Janine Baumann, Joanna Piekarski, Jo-Anne Dalton, Joy Johnson-Green, Karen Wood, Marcia Bruce, Maria Santana, Matt Mayer, Meghan Gould, Michael Kobor, Michelle Flowers, Michelle Haywood, Michelle Koerner, Nancy Parker, Nazeem Muhajarine, Paul Fairie, Rabea Chrishti, Robert Perry, Sarah Merrill, Shellie Pociuk, StephanieTaylor, Steve Cole, Tim Murphy, Tmira Marchment, Virginia Xavier, Zahra Shajani, Zoe West

**Affiliations:** 1 Owerko Centre at the Alberta Children’s Hospital Research Institute, University of Calgary, Calgary, AB Canada; 2 Owerko Centre at the Alberta Children’s Hospital Research Institute, Child Development Centre, 3rd Floor, 3820 24 Avenue NW, University of Calgary, Calgary, Alberta T2N 1N4 Canada; 3 Athabasca University, Calgary, AB Canada; 4 Ottawa Hospital Research Institute, Ottawa, ON Canada; 5 University of Calgary, Calgary, AB Canada; 6 University of Manitoba, Winnipeg, MB Canada; 7 McGill University, Montreal, QC Canada; 8 CUPS Calgary, Calgary, AB Canada; 9 Alberta Health Services, Calgary, AB Canada; 10 University of Alberta, Edmonton, AB Canada; 11 The University of British Columbia, Vancouver, BC Canada; 12 Marymound Inc., Winnipeg, MB Canada; 13 Elizabeth Fry Society, Ottawa, ON Canada; 14 Sonshine Community Services, Calgary, AB Canada; 15 Alberta SPOR Support Unit, University of Alberta, Edmonton, AB Canada; 16 Discovery House Family Violence Prevention Society, Calgary, AB Canada; 17 University of Saskatchewan, Saskatoon, SK Canada; 18 Family Service Saskatoon, Saskatoon, SK Canada; 19 Steinbach Family Resource Centre, Steinbach, MB Canada; 20 Regina Transition House, Regina, SK Canada; 21 BC Children’s Hospital Research Institute, Vancouver, BC Canada; 22 Saskatchewan Centre for Patient-Oriented Research (SCPOR), Saskatoon, Sk Canada; 23 Elizabeth House Calgary, Calgary, AB Canada; 24 Family Service Regina, Regina, SK Canada; 25 University of California (Los Angeles), Los Angeles, CA USA; 26 Alberta Innovates, Calgary, Canada; 27 SOFIA House, Regina, SK Canada

**Keywords:** Study protocol, ATTACH^TM^, EIH type 2 clinical trial, Quasi-experimental design, Parenting program, Reflective function, Parent-child interaction, Child development, Normalization process theory

## Abstract

**Background:**

Children’s exposure to toxic stress (e.g., parental depression, violence, poverty) predicts developmental and physical health problems resulting in health care system burden. Supporting parents to develop parenting skills can buffer the effects of toxic stress, leading to healthier outcomes for those children. Parenting interventions that focus on promoting parental reflective function (RF), i.e., parents’ capacity for insight into their child’s and their own thoughts, feelings, and mental states, may understand help reduce societal health inequities stemming from childhood stress exposures. The Attachment and Child Health (ATTACH^TM^) program has been implemented and tested in seven rapid-cycling pilot studies (*n* = 64) and found to significantly improve parents’ RF in the domains of attachment, parenting quality, immune function, and children’s cognitive and motor development. The purpose of the study is to conduct an effectiveness-implementation hybrid (EIH) Type II study of ATTACH^TM^ to assess its impacts in naturalistic, real-world settings delivered by community agencies rather than researchers under more controlled conditions.

**Methods:**

The study is comprised of a quantitative pre/post-test quasi-experimental evaluation of the ATTACH^TM^ program, and a qualitative examination of implementation feasibility using thematic analysis via Normalization Process Theory (NPT). We will work with 100 families and their children (birth to 36-months-old). Study outcomes include: the Parent Child Interaction Teaching Scale to assess parent-child interaction; the Parental Reflective Function and Reflective Function Questionnaires to assess RF; and the Ages and Stages Questionnaire – 3rd edition to examine child development, all administered pre-, post-, and 3-month-delayed post-assessment. Blood samples will be collected pre- and post- assessment to assess immune biomarkers. Further, we will conduct one-on-one interviews with study participants, health and social service providers, and administrators (total *n* = 60) from each collaborating agency, using NPT to explore perceptions and experiences of intervention uptake, the fidelity assessment tool and e-learning training as well as the benefits, barriers, and challenges to ATTACH^TM^ implementation.

**Discussion:**

The proposed study will assess effectiveness and implementation to help understand the delivery of ATTACH^TM^ in community agencies.

**Trial registration:**

Name of registry: https://clinicaltrials.gov/. Registration number: NCT04853888. Date of registration: April 22, 2021.

**Supplementary Information:**

The online version contains supplementary material available at 10.1186/s12887-022-03439-3.

## Administrative information

Note: the numbers in curly brackets in this protocol refer to SPIRIT checklist item numbers. The order of the items has been modified to group similar items (see http://www.equator-network.org/reporting-guidelines/spirit-2013-statement-defining-standard-protocol-items-for-clinical-trials/).

**Table:** 

**Section/item**	**Item No**	**Description**
**Administrative information**		
Title	#1	Study Protocol for Attachment & Child Health (ATTACH^TM^) Program: Promoting Vulnerable Children’s Health at Scale
Trial registration	#2a and #2b	https://clinicaltrials.gov/ Registration number: NCT04853888. Date of registration: April 22, 2021.
Protocol version	#3	March 1, 2021, Version 1
Funding	#4	This study is funded by the Canadian Institute of Health Research (CIHR) Strategies for Patient-Oriented Research (SPOR) Innovative Clinical Trial Multi-Year Grant.
Roles and responsibili-ties	#5a	Lubna Anis, Owerko Centre at the Alberta Children’s Hospital Research Institute, University of Calgary, Calgary, AB. Role: Postdoctoral Fellow. Nicole Letourneau, Owerko Centre at the Alberta Children’s Hospital Research Institute, University of Calgary, Calgary, AB. Role: Principal Investigator. Kharah Ross, Owerko Centre at the Alberta Children’s Hospital Research Institute, University of Calgary, Calgary, AB, Athabasca University, AB. Role: Co-investigator. Martha Hart, Owerko Centre at the Alberta Children’s Hospital Research Institute, University of Calgary, Calgary, AB. Role: Project Lead. Ian D Graham, University of Ottawa, Ottawa, ON. Role: Member of the Parent Engagement Committee (PEC) and Community Engagement Committee (CEC), defined below. Simone Lalonde, University of Calgary, Calgary, AB. Role: Member of the PEC. Suzanna Varro, University of Calgary, Calgary, AB. Role: Member of the PEC. Alanna Baldwin, University of Manitoba, Winnipeg, MB. Role: Member of the SPOR Committee. Angela Soulsby, University of Calgary, Calgary, AB. Role: Member of the PEC. Annette Majnemer, McGill University, Montreal, QC. Role: Member of the SPOR Committee. Carlene Donnelly, CUPS Calgary, Calgary, AB. Role: Member of the CEC. Caroline Piotrowski, University of Manitoba, Winnipeg, MB. Role: Member of the PEC and the CEC. Carrie Collier, Alberta Health Services. Role: Member of the PEC and the CEC. Cliff Lindeman, University of Alberta, Edmonton, AB. Role: Member of the Strategy for Patient Oriented Research (SPOR) Committee, defined below. Dan Goldowitz, T he University of British Columbia, Vancouver, BC. Role: Member of the SPOR Committee. Dawn Isaac, Marymound Inc., Winnipeg, MB. Role: Member of the CEC. Denise Thomson, University of Alberta, Edmonton, AB. Role: Member of the SPOR Committee. Diane Serré, Elizabeth Fry Society, ON. Role: Member of the CEC. Elisabeth Citro, Sonshine Community Services, Calgary, AB. Role: Member of the CEC. Gabrielle Zimmermann, Alberta SPOR Support Unit, University of Alberta, Edmonton, AB. Role: Member of the Strategy for SPOR Committee. Harold Pliszka, Discovery House Family Violence Prevention Society, Calgary, AB. Role: Member of the PEC and the CEC. Jackie Mann, University of Saskatchewan, Saskatoon, SK. Role: Member of the SPOR Committee. Janine Baumann, Family Service Saskatoon, Saskatoon, SK. Role: Member of the CEC. Joanna Piekarski, Alberta Health Services. Role: Member of the CEC. Jo-Anne Dalton, Steinbach Family Resource Centre, Steinbach, MB. Role: Member of the CEC. Joy Johnson-Green, Sonshine Community Services, Calgary, AB. Role: Member of the CEC. Karen Wood, University of Saskatchewan, Saskatoon, SK. Role: Member of the PEC and the SPOR Committees. Marcia Bruce, University of Calgary, Calgary, AB. Role: Member of the PEC. Maria Santana, University of Calgary, Calgary, AB. Role: Member of the SPOR Committee. Matt Mayer, University of Calgary, Calgary, AB. Role: Member of the PEC. Meghan Gould, Regina Transition House, Regina, SK. Role: Member of the CEC. Michael Kobor, BC Children’s Hospital Research Institute, Vancouver, BC. Role: Co-investigator. Michelle Flowers, Saskatchewan Centre for Patient-Oriented Research (SCPOR). Role: Member of the SPOR Committee. Michelle Haywood, Elizabeth House Calgary, Calgary, AB. Role: Member of the CEC. Michelle Koerner, Discovery House Family Violence Prevention Society, Calgary, AB. Role: Member of the CEC. Nancy Parker, Marymound Inc., Winnipeg, MB. Role: Member of the CEC. Nazeem Muhajarine, University of Saskatchewan, Saskatoon, SK. Role: Member of the SPOR Committee. Paul Fairie, University of Calgary, Calgary, AB. Role: Member of the SPOR Committee. Rabea Chrishti, University of Calgary, AB Role: Member of the CEC. Robert Perry, CUPS Calgary, Calgary, AB. Role: Member of the CEC. Sarah Merrill, The University of British Columbia, Vancouver, BC. Role: Co-investigator Shellie Pociuk, Family Service Regina, Regina, SK. Role: Member of the CEC. StephanieTaylor, Regina Transition House, Regina, SK. Role: Member of the CEC. Steve Cole, University of California (Los Angeles), Los Angeles, CA. Role: Co-investigator. Tim Murphy, Alberta Innovates Role: Member of the SPOR Committee. Tmira Marchment, SOFIA House, Regina, SK. Role: Member of the CEC. Virginia Xavier, University of Calgary, Calgary, AB. Role: Member of the PEC and the CEC. Zahra Shajani, University of Calgary, Calgary, AB. Role: Member of the CEC. Zoe West, Elizabeth Fry Society, Ottawa, ON. Role: Member of the CEC.
	#5b	CIHR funded investigator initiated innovative trial; N. Letourneau (Principal Investigator) Contact: Nicole.letourneau@ucalgary.ca
	#5c	The study is an investigator-initiated innovative trial. The funders had no role in the study design and data collection, data analysis, and interpretation of data, or in writing the protocol.
	#5d	An ATTACH^TM^ Team Executive Committee, chaired by N. Letourneau will oversee all project activities, with members including Patient/ Principal Knowledge User (K. Ross), CEC co-chairs (C. Collier and H. Pliszka) and PEC co-chairs (S. Lalonde and S. Varro), Project Manager (M. Hart) and other investigators (Epigenetics: M. Kobor & S. Merrill; Gene Expression: K. Ross and S. Cole; Data Analysis and Management: H. Ntanda). Coordinating Centre: Owerko Centre at the Alberta Children’s Hospital Research Institute, University of Calgary, Calgary, AB.

## Introduction

### Background and rationale {6a and 6b}

Children’s exposure to toxic stress (e.g., parental depression, violence, poverty) predicts developmental problems (e.g., cognitive, motor), behavioural disturbances, physical health problems, and years of life lost to disability, resulting in health care system burden [1–4]. Effective parenting skills can buffer the effect of toxic stress exposure on child developmental and health outcomes [5]. Interventions that focus on supporting effective parenting in the face of parental depression, violence, and other threats to well-being are effective strategies to support healthy child development [6, 7]. Furthermore, parenting interventions that focus on reflective function (RF), that is, parental capacity to understand and thus regulate their own and their child’s thoughts, feelings, and mental states, [8–10], have great potential to improve developmental outcomes of children exposed to toxic stress. Indeed, RF is essential for high quality parent-child relationships and parent-child attachment [11], child development, and health. Specifically, there is evidence for improvement in: cognition [12], communication [13], mental [14], and immune health [15]. Effective early interventions focusing on parental RF, such as the Attachment and Child Health (ATTACH^TM^) [10, 16] program, may help reduce societal health inequities stemming from childhood stress exposures [9, 17].

ATTACH^TM^ is a psychoeducational parenting program delivered in community agencies that serve families with preschool-aged children affected by toxic stress [18]. It was tested in seven rapid-cycling pilot studies with small sample sizes (*n*’s = 10–20 per pilot). Pilots were guided by the IDEAS (Innovate, Develop, Evaluate, Adapt, Scale) Framework^TM^ [18], an innovative clinical trial approach that reduces time from program development to implementation, while ensuring rigour in evaluation [18, 19]. In two phases, we conducted randomized control trial (RCT) and quasi-experimental (QE) pilot studies. In the first, phase, three pilot studies revealed that ATTACH^TM^ significantly improved parent-child relationship quality (*d* = .34–1.5) [9] parents’ RF scores (*d* = .51–2.0) [10], and children’s cognitive and motor development(*d* = .98–2.3; all *p*’s < .05) [9, 10]. These findings led the Harvard Center on the Developing Child to recognize and fund ATTACH^TM^ as a Frontiers of Innovation project (see https://developingchild.harvard.edu/innovation-application/frontiers-of-innovation/), supporting phase two. In the four pilot studies that followed, ATTACH^TM^ significantly improved parent-child interaction quality (*d* = .41–1.5), parental reflective function (*d* = 1.2–1.4), attachment security (eta^2^ = .30–.47), child development (*d* = .47–1.4; all *p*’s < .05) and immune cell gene expression [20, 21]. While these preliminary results are promising, whether ATTACH^TM^ is as effective when delivered by trained agency health and social service providers rather than researchers (as in the first seven pilot studies), in naturalistic community settings remain to be seen.

Further, to our knowledge only two studies of parenting interventions, including our pilot research [20, 22] have shown changes in systemic inflammation in children exposed to toxic stress. Whether ATTACH^TM^ affects novel biomarkers (i.e., immune cell gene expression, DNA methylation) in a larger sample is not known. Addressing this gap offers the rare opportunity to examine how parent-child relationships could buffer impacts of toxic stress on immune cell regulation, via gene expression and epigenetics, which is considered a research priority [23, 24]. Through integrated Knowledge Translation (iKT) and Patient Engagement (PE) approaches [25], where patients,^1^ health and social service providers, and health system administrators are partners [26–28], and along with our funder Canadian Institutes of Health Research (CIHR) Strategy for Patient-Oriented Research (SPOR) collaborators [e.g. Alberta SPOR Support Unit, Saskatchewan Centre for Patient-Oriented Research (SCPOR)], this team proposes to undertake an effectiveness-implementation hybrid (EIH), Type II study [29] of ATTACH^TM^, with co-primary objectives in clinical intervention effectiveness and implementation strategy evaluation [29]. The co-primary objectives evaluate clinical intervention effectiveness and implementation strategy feasibility in naturalistic, real-world, settings, as delivered by community agencies.

### Impact of toxic Stress on parent-child relationships and child development and health

An estimated 25–33% of Canadian and American preschool-aged children are raised in families with at least one toxic stressor [18, 30]. Previous research has found that the developmental costs of exposure to toxic stress are high. Maternal depression is well-known to negatively impact child development [31–33]. Children aged 12 to 24 months exposed to postpartum depression have significantly reduced cognitive development characterized by lower communication scores [34] and 2 to 6 year olds have reduced cognitive (e.g., communication, problem-solving, personal, social) and motor development [35]. Six to 18 month-old children exposed to family violence are at greater risk for developmental problems [23, 24] and specifically lower cognitive and motor domain scores [36]. One to 3 year-old children exposed to family violence also experience cognitive and motor delays, specifically reduced problem-solving and fine motor skills respectively [37]. Similar impacts of poverty on children’s developmental attainment have also been observed [38, 39]. Toxic stress also affects physical health and the immune system, as associated with higher systemic inflammation in adulthood [40–42] and inflammatory disease risk (e.g. cardio-metabolic disease, cancer, asthma, depression) [43, 44]. Recently, inflammation has been proposed as the intermediary between toxic stress exposure and children’s cognitive and mental developmental outcomes [45, 46].

A troubling additional consequence of toxic stress exposure is compromised parent-child relationships, characterized by reduced parental sensitivity/responsiveness and insecure parent-child attachment [47, 48]. A systematic review [49] revealed that reduced parental sensitivity/responsiveness undermines children’s attachment security, development, and health, especially in cognitive and motor domains. Findings held across a diverse range of cultures [49], including Canadian Indigenous peoples [50]. The review concluded that less sensitive/responsive parental behaviors and cognitions are consistent with the toxic stressor of addiction (e.g., due to addiction-related inconsistency in infant care, changing mood) [51] or depression (e.g., due to symptoms of fatigue, reduced concentration) [32] that induce parents to miss and thus fail to respond to their children’s cues [52, 53]. In general, children’s development is negatively impacted when parents fail to: (1) recognize and respond to children’s cues that signal needs and (2) regulate their children’s mental and emotional states [48]. Both are targets of the ATTACH^TM^ intervention.

Toxic stress also undermines children’s attachment security, which develops in infancy from the emerging expectation that the infant’s basic needs for soothing, comfort, and protection from danger will be met (or not) by their parent [54–57]. Insecure attachment predicts a well-studied host of developmental problems [12, 49–54], including cognitive challenges [58, 59], and an emerging literature on physical health problems linked to inflammation [15, 60]. High quality parent-child relationships, characterized by secure attachment at 12 months of age, predicted reduced risk for inflammatory disease *30 years later* [15]. Further, research from our team found that high quality maternal-child relationships characterized by sensitivity/responsiveness buffered the effect of toxic stress on child immune system activity [61]. Reduced parent-child relationship quality thus appears to leave a long-lasting imprint on children’s development and health.

### Parent-child relationships and parental reflective function (RF)

High quality parent-child relationships, characterized by sensitivity/responsiveness to children’s states and emotions, are in part the result of parental RF [62], Parental RF is necessary to perceive and respond to a child’s cues for comfort, soothing, or exploration [63–65], and is characteristic of optimal parent-child relationships. For example, a parent who is unable to recognize their child’s fear of separation is likely to fail both to reassure the child that they will return and to regulate their child’s stress response. Parents’ experiences of depression, violence, and/or addictions) [66–68] and past traumas (e.g. histories of unresolved grief, emotional, physical, or sexual abuse) [69, 70] predict parents’ negative and distorted representations of reality and frightened, frightening, or dissociated behaviors during interactions with their young children [66–70]. These parents are at risk for reduced RF, adverse parent-child relationship quality and their children are at risk for insecure attachment and long-lasting negative development and health outcomes [5, 18]. These parents are the target of the ATTACH^TM^ intervention.

### Parental RF focused interventions

Three meta-analyses reveal that high parental RF can buffer the negative effects of toxic stress [11, 71, 72]. Our meta-analysis demonstrated that RF is modifiable by intervention, and that RF-focused parenting interventions predict improvements in parent-child relationship quality in families affected by a variety of toxic stressors [11]. Preserving optimal RF when experiencing toxic stress allows parents to attribute affective states to their children and respond to meet their children’s needs, thus promoting healthy parent-child relationships [63, 65, 73]. Targeting parental RF improvement is thus a promising way to tackle the impacts of toxic stress on vulnerable children’s development. With respect to immune health, emerging evidence suggests that parenting interventions can reduce systemic inflammation [22] and pro-inflammatory immune cell epigenetic programing [74] in children 10 years after intervention. Whether RF-focused interventions like ATTACH^TM^ affects children’s inflammation is unknown.

### ATTACH^TM^ pilot studies using innovative clinical trial and iKT approaches

The focus of ATTACH^TM^ on RF as well as the design of key intervention components were determined in collaboration with knowledge users including community health and social service agency administrators and health and social service providers. First, they pointed out that the most effective parenting programs tend to include a focus on RF [11, 75], and that these programs are few in number, thus RF became the target of ATTACH^TM^. Second, collaborators noted that existing RF-focused parenting programs typically involve months or years of psychotherapy provided by expensive clinical psychologists or psychiatrists [76], rendering these approaches cost-prohibitive and thus unrealistic in community settings. ATTACH^TM^ was thus designed to be a shorter intervention and simple enough to be administered by professionals with an undergraduate education in a health-related field, typical of partner agency staff. Third, many parenting programs tend to be designed for a narrow patient population (e.g., those affected by maternal addiction or depression, but not both) [77], making such programs difficult to administer in agencies serving more complex clients. Thus, ATTACH^TM^ was designed to help complex patients by targeting rapid learning of parental RF skills via practice, from which many could benefit. Finally, parenting programs often do not incorporate co-parents, defined as individuals who are a main source of parenting support (e.g., other parent, partners, grandparents, friends) [78, 79]. As a result, the inclusion of co-parents in ATTACH^TM^, was guided by health and social service providers /knowledge users’ suggestion [80].

Since completion of the seven pilot studies, we made additional changes, based on feedback from collaborators. First, we were advised to address the broad range of family structures served in partners’ agencies including both sexes/self-reported genders of parents. While mothers and children were the focus in pilots, ATTACH^TM^ has now been adapted to the primary child caregiver (hereafter referred to as “parent”, whether mother, father, grandparent, foster carer, cis-or trans-gender, etc.) and their source of co-parenting support. As a result, ATTACH^TM^ will be more applicable across a diversity of family units and more realistic for implementation in community agencies. Second, we were advised by health and social service providers /knowledge users to make the ATTACH^TM^ materials more ethnically diverse. We have since changed the manual images to reflect a variety of cultures and names. Finally, we were advised by agency health and social service providers to make the training more accessible. To respond to this suggestion, we developed an on-line platform for training and accrediting health and social service providers to deliver ATTACH^TM^ independently in their agencies (beta version at https://attach.teachable.com) and resulted in trademarking the ATTACH^TM^ name. In summary, our innovative clinical trial and iKT approaches resulted in targeting the ATTACH^TM^ intervention design to address key agency needs including cost-effective programming (shorter duration) that is aligned with staff education level, broadening the focus to include a variety of at-risk parents and their co-parents, and now to include parents and co-parents of any description. We also responded to suggestions to make materials more ethnically diverse, and to develop an on-line training platform. While our iKT approaches involved many collaborators, we did not systematically engage with parents in intervention design. We anticipate the benefits of more systematic and sustained parent “patient” (see footnote 1) engagement along with iKT approaches in our proposed project.

### Objectives {7}

Building on input from community health and social service agency administrators and health and social service providers, our completed pilot work, and the urgent need for RF interventions to address impacts of toxic stress on vulnerable children in the community, this team aims to address two co-primary objectives, Thus, our EIH design (Type II) [29] addresses co-primary objectives in Effectiveness of Clinical Intervention (Objective 1) and Feasibility of Implementation Strategy (Objective 2). We will complete this work with the assistance of our Community Engagement Committee (CEC), Patient Engagement Committee (PEC) and Support for Patient Oriented Research (SPOR) Committee.

#### Objective 1 (quantitative arm)

With quasi-experimental methods we will (1a) Evaluate ATTACH^TM^ impacts on parent-child relationship quality (primary outcome), parental reflective function, and child development; (1b) determine whether ATTACH^TM^ is equally effective across patient populations (and for whom it works best/worst); (1c) evaluate whether ATTACH^TM^ impacts immune biomarkers indicative of inflammatory disease risk, i.e. gene expression and DNA methylation; and (1d) evaluate the long-term impact of ATTACH^TM^ (3 months post-intervention).

#### Objective 2 (qualitative arm)

We will (2a) Evaluate implementation benefits, facilitators, barriers, and challenges from patients’, health and social service providers’, and health system administrators’ perspectives; and (2b) evaluate the utility of our new ATTACH^TM^ e-learning platform.

### Trial design {8}

This EIH type 2 [29] study is a quasi- experimental evaluation of the ATTACH^TM^ program and implementation feasibility [75, 76]. **Objective 1: ATTACH**^**TM**^ **Impacts**, will be addressed via a quasi-experimental design to closely approximate service delivery models in agencies that do not typically employ control groups. Moreover, a RCT design, even employing wait-list controls was deemed unacceptable and even unethical by parents, health and social service providers, and health system administrators in engagement activities surrounding the preparation of this proposal. **Objective 2: ATTACH**^**TM**^ **Implementation Feasibility,** will be addressed via Normalization Process Theory (NPT) to understand factors that promote or inhibit interventions from being embedded in practice [75–77]. Developed in response to recognition of the large gap between intervention development and intervention use in health care—the ‘know-do gap’—, NPT is intended to uncover factors that interfere with the routine or “normal” incorporation of interventions into health care [78]. NPT will guide qualitative semi-structured interviews and analysis exploring patients’, health and social service providers’, and health system administrators’ perceptions and experiences of the benefits, facilitators, barriers, and challenges to ATTACH^TM^ implementation in community agencies.

## Methods: participants, interventions, and outcomes

### Participants

#### Objective 1

##### Parents, co-parents, and children

Those participating families (both mother and mother-identified co-parenting support person) participating in services or programs in the partner agencies are our study population.

#### Objective 2

Parents, Co-Parents, as well as their health and social service providers. Those who have completed the ATTACH^TM^ program or have been responsible for its delivery in partner agencies.

##### Stakeholder engagement

Building on our successful iKT efforts in our pilot work, we will systematically build stakeholder engagement into our study design. Key stakeholders including the Parent Engagement Committee (PEC), the Strategy for Patient Oriented Research (SPOR) Committee, and the Community Engagement Committee (CEC) will be involved in the proposed research. The PEC is defined as a team of individuals who are involved in co-building the intervention, with parents invited to partner and provide feedback on intervention implementation and evaluation. Parents are defined broadly, inclusive of individuals with personal experience of an issue; who provide care or support to those affected by an issue; and with indirect experience of an issue, through family or friend experience or professional practice. The SPOR Committee is defined as a team of individuals who will guide and help oversee the research on the use of patient (parent) and other stakeholder engagement and integrated knowledge translation activities. Also, the CEC is defined as a team of health and social service providers, and administrators in agencies who will have the chance to participate in the project roll-out, participate in interviews as participants, learn about implementation barriers and facilitators, and have opportunities to take part in ATTACH^TM^ training and ultimately independent delivery and evaluation.

### Study setting {9}

#### Objective 1

Settings include our partner agencies in Alberta, Manitoba, and Saskatchewan that serve families experiencing toxic stress We will work with 100 new families from an initial 4 community agencies (Calgary Urban Project Society, Calgary, AB, Discovery House Women’s Shelter, Calgary, AB, Sonshine Community Centre, Calgary AB, and Steinbach Family Resource Centre, Steinbach, MB) and additional community partners (Elizabeth House, Calgary, AB, Regina Transition House, Regina, SK, Marymound, Winnipeg, MB, SOFIA House, Regina, SK, Elizabeth Fry Society, Ottawa, ON) are dissemination targets. The initial 4 implementation sites have a successful history of delivering programs focused on promoting health and development of children in families affected by toxic stress and provide an optimal testing ground for the ATTACH^TM^ intervention. Currently, these agencies do not offer any RF-based parenting programs. The ATTACH^TM^ program will be added on to their existing services or programs. and will disseminate knowledge to additional partners over the course of the study. **Objective #2:** For the qualitative component of the study, we will interview study participants who complete the ATTACH^TM^ intervention in the initial 4 community agencies (Calgary Urban Project Society, Calgary, AB, Discovery House Women’s Shelter, Calgary, AB, Sonshine Community Centre, Calgary AB, and Steinbach Family Resource Centre, Steinbach, MB), as well as their health and social service providers and agency administrators at the same partner agencies, using NPT [75, 76, 79] .

### Eligibility criteria {10}

#### Objective #1

The study inclusion criteria include: (1) parents with children between birth to 32 months of age (our age ceiling is 36 months, based on selection of age-appropriate tools for assessing children’s health and development); (2) parents who agree to participate in the ATTACH^TM^ program consisting of 10 weeks of additional, concurrent, one-hour per week parent training sessions; (3) parents who agree to bring a co-parent for 2 of the 10 sessions (when possible); (4) parents who agree to the dried blood sample collection to assess immune biomarkers (in Calgary agencies only, due to logistics issue related to blood sample collection, storage, and shipment).

#### Objective #2

Participants (i.e., parents, co-parents, community health and social service agency administrators and health and social service providers) must be adults (18 years of age or older), and English speaking and reading, and knowledgeable or experienced in parenting programs.

### Intervention {11a, 11c and 11d}

ATTACH^TM^ intervention sessions include three components:

- Parent-child interactions are videotaped during 3–5 minutes of free-play for review and discussion. During the video feedback, the ATTACH^TM^ facilitator explores the parents’ perceptions of themselves and their children, and maximizes opportunities to practice RF (e.g., parents are asked to consider what may be happening with her thoughts and feelings and with her child’s thoughts and feelings during a shared smile in the videotaped interaction).
- A hypothetical, mildly stressful situation is discussed. Hypothetical situations encourage imagining others’ thoughts and feelings in a common daily life, such as during dinner when the child throws food on the floor.
- Day-to-day real-life stressful situations are discussed. Real-life stressful situation reviews revolve around parents’ and others’ thoughts and feelings as the stressful situation is described in detail. When co-parents are present, only the free-play videotaped interactions and hypothetical situations are examined. To avoid any potential conflict, real-life stressful situations are excluded from co-parent sessions.

Throughout, ATTACH^TM^ interventionists bolster parents’ RF through questioning and maintaining a curious stance about parents’, children’s and others’ thoughts and feelings. Once the therapeutic relationship is established (after approximately 6 one-on-one sessions of therapy), parents invite their co-parenting support person (e.g., father, mother, relatives, friends, or other support person) to 2 sessions, typically at sessions 7 and 9, spaced 2 weeks apart. At least 2 interventionists will be trained in ATTACH^TM^ at each agency using online modules and in-person practice that takes a total of 20 hours.

### Outcomes {12}

Parent-Child Relationship Quality will be measured with the Parent-Child Interaction Teaching Scale (PCITS) [81]. The PCITS is an observational binary measure of relationship quality in an everyday teaching situation, designed for children 36 months or younger. It is considered the gold standard for the assessment of parent-child relationship quality. The PCITS consists of 73 items categorized into six subscales including parental sensitivity to cues, responsiveness to distress, growth fostering, and cognitive growth fostering, child clarity of cues and responsiveness to parent as well as parent total, child total, and parent-child total scores. The scales’ reliability and validity are well established and was observed to be a strong measure of intervention impact in our pilot studies . The observation typically takes 5 minutes to administer and is videotaped to enhance the accuracy of data coding. ATTACH^TM^ interventionists who are health and social service providers in agencies, will be trained to administer the videotaping. Colleagues from the University of Washington will code all videos. During the pilot studies, they retained > 95% intra-rater reliability on 10% of recoded videos and reliable at the 90th percentile with the Canadian study team. Coders are supervised by PI Letourneau who has been a certified PCITS trainer since 1996 and has kept up annual certification. A protocol for data collection was developed as part of the pilot studies and discussions with patients revealed that the assessment is feasible and acceptable.

#### Secondary outcomes. Reflective function (RF)

Two measures of RF will be employed, one to assess general RF and the other to assess parental RF. General RF is distinct from parental RF as the concept of parental RF is more parent–child relationship-specific [82]. The Parental Reflective Function Questionnaire (PRFQ) [80] is an 18-item measure of parental RF, with subscales assessing (1) certainty about mental states and (2) interest and curiosity about mental states. Higher scores indicate higher levels of RF. The PRFQ has good internal consistency (.7–.84) and takes 5 minutes to complete refs. The Reflective Functioning Questionnaire (RFQ) [83] is an 8-item measure of RF that provides a total score for RF. Created by Peter Fonagy, the measure has acceptable reliability and validity and takes less than 5 minutes to complete. Pilot testing revealed the PRFQ and RFQ each detected intervention impacts and were acceptable to patients. In our other work [82], we showed that scores on the RFQ and PRFQ were significantly associated with the gold standard Parental Development Interview coded with Fonagy’s 11-point scale. Given the gold standard requires 1–2 hours per patient interview, 3–4 hours for transcription, followed by 2 hours coding per interview, the use of the RFQ and PRFQ significantly reduces both patient burden and cost of administration and are feasible to implement in agencies.

We will employ the Ages and Stages Questionnaire-3rd Edition (ASQ-3) [84] to assess child development in cognitive and motor domains. The cognitive measures include communication, problem solving, and personal-social skills development, while motor measures include both gross and fine motor development. The ASQ-3 is a series of parent-completed questionnaires for age groups ranging from 1 to 66 months, asking six questions in each domain assessing children’s abilities to undertake age-appropriate tasks. Summing items in each domain provides total scores (maximum 60) with higher scores indicating more optimal development; cut-offs are provided to indicate risk for developmental delay in each domain, appropriate to each age group. The ASQ-3 is typically administered in community agencies, thus both agencies and our pilot patients found this measure acceptable and feasible. The ASQ-3 is found to have strong internal consistency reliability (between 0.82–0.88), sensitivity of 86% and a specificity of 85% [85] and is used to identify children at risk for developmental disorder [86].

Dried blood spots (DBS) for assessment of immune biomarkers will be collected by either ATTACH^TM^ interventionists/agency health and social service providers or the research team (agency preference), pre- and post-assessment (only; no 3 month delayed post-intervention assessment), to assay children’s immune cell gene expression (mRNA) [87] and epigenetics (DNA methylation) [88]. DBS collection protocols to reduce distress/discomfort of children were pilot tested with 20 families. While the DBS pilot was optional and parents’ choice, *all children* (*n* = 20) provided blood samples pre- and post-assessment, with minimal distress in children or concerns expressed by parents. Our consultation with agency partners dictates that the DBS will remain optional to avoid deterring patients from participating in ATTACH^TM^. However, our high pilot uptake rate (100%) suggests we will have a sufficient sample. Briefly, a finger is pricked and drops of blood are collected onto specially prepared filter paper cards.

DBS collection is less invasive than traditional venipuncture techniques and is specifically designed for field use [89]. Sex and gender based analysis will also be conducted [90].

#### Objective 2: ATTACH^TM^ implementation feasibility

Using NPT in implementing complex interventions requires the developing an interview guide to address four key concepts: coherence, cognitive participation, collective action, and reflexive monitoring of the intervention [78]. In finalizing the interview guide and developing the interview processes (e.g., home or clinic setting, telephone/skype, or in-person), Patient Engagement Committee (PEC) and Community Engagement Committee (CEC) members will review, provide feedback, and make decisions about interview guide content. The interview guide will be pilot tested before use. Interviews will be audio-recorded and transcribed verbatim with appropriate privacy protections in place to guard participant identity and personal information, followed by conducting qualitative thematic analysis [91].

### Participant timeline {13}

#### Objective 1

##### Pre-assessment

The ATTACH^TM^ program begins with administering pre-assessment data collection. This is essential to evaluate the ongoing effectiveness of ATTACH^TM^. The participants will be asked to provide observational, questionnaire, and biological data.

##### ATTACH^TM^ reflective function intervention

The ATTACH^TM^ Reflective Function Intervention can begin after the pre-assessment sessions are completed–usually the following week. ATTACH^TM^ intervention sessions include three components, described above in {11}:

##### Post-assessment phase

The ATTACH^TM^ intervention must be complete before post-assessment which includes parents’ providing observational, and questionnaire data. Dried blood spots (DBS) will be collected by either ATTACH^TM^ interventionists/ agency health care professionals or the research team (agency preference), pre- and post-assessment (only; no 3-month delayed post-assessment), to assay children’s immune cell gene expression (mRNA) and epigenetics (DNA methylation).

##### Delayed post-assessment

Three months after the post-assessment data collection is complete, parents will again be asked to evaluate the ongoing effectiveness of ATTACH provide observational, and questionnaire to evaluate the ongoing effectiveness of ATTACH^TM^.

#### Objective 2

NPT interviews will begin as soon as the first family completes the ATTACH^TM^ Reflective Function Intervention Phase and will continue until data saturation is attained, i.e., the degree to which new data repeats or is redundant with what was expressed in previous data [92]. We will employ a stopping rule: Data collection in a category (patient, health and social service providers, and health care administrator) will cease when three interviews in a row offer less than 5% new information (i.e., only one new code to one question of 19 in interview guide offers new information).

### Sample size {14}

#### Objective 1

##### Quantitative component

We will recruit until we have completed all study components with 100 new families, a sufficient *n* to detect minimum *d* = .34 for pre- /post- assessment differences in parent-child relationship quality. From discussions with agency health and social service providers and administrators, it will be feasible to recruit 30 families (including parents, co-parents, and children) from each agency, from patients currently seeking service, to retain 20 of them for 3 months postintervention for follow-up (any longer was deemed unrealistic given potential for patient relocation). Specifically, iKT and end-of-grant dissemination will be planned in partnership with agency health and social service providers and administrators.

#### Objective 2

##### Qualitative interviews guided by NPT

From discussions with agency administrators, it will be feasible to recruit 20 patients, 20 health and social service providers and 20 administrators (total *n* = 60) for interviews. However, recruitment and data collection will continue only until theoretical data saturation.

### Recruitment {15}

#### Objective 1

##### **ATTACH**^TM^**impacts**

Every partner agency (including additional agencies) will recruit 2–5 staff members for ATTACH^TM^ training to become the ATTACH^TM^ facilitators. To partake in the ATTACH^TM^ parenting program, participants will be identified through partner agencies. We will intervene with up to 150 parents and their birth to 36-month-old children to retain 100, from 5 community agencies. Parents will be recruited from rosters of patients currently seeking service. The ATTACH^TM^ information sheets and brochures will be posted on implementation sites. Their current and interested clients will be our potential participants. Their staff will assist with recruitment as participants seek their services in routine care.

#### Objective 2

##### ATTACH^TM^ implementation feasibility

Patients, health and social service providers and administrators from each of the five initial agencies will be recruited via convenience sampling methods. We anticipate 4 patients, 4 health and social service providers, and 4 decision makers will be recruited for interviews from each agency, for a total of 20 participants in each category.

## Methods: data collection, management, and analysis

### Data collection methods {18a, 18b)

#### Objective 1: ATTACH^TM^ impacts

We have engaged with our agency partners on the collection of parents’ and children’s demographic information, descriptive data on exposure to toxic stressors, outcome measures, and covariates. To reduce participant burden, data on demographic variables will be obtained from agency administrative records as much as possible (e.g., ethnicity, sex, and gender, first language, marital status, education, employment, number of children, and age of patients and children) at pre-assessment. To further reduce burden, many measures have been selected from intake data collection already conducted in agencies, e.g. ASQ-3 [84]. Covariate measures will primarily be those already given at patient intake and that focus on toxic stress exposures, i.e. depressive symptoms [Edinburgh Depression Scale (EDS) [93, 94], a 10-item self-report tool to measure depression and exhibits sensitivity of 66.7–69% and specificity of 67.7% and takes 5 minutes to administer], family violence [Revised Conflict Tactics Scale-Short Form (CTS2-SF) [95], a 20-item questionnaire with internal consistency of 0.79–0.95 and takes 3 minutes to complete], addictions [Alcohol, Smoking and Substance Involvement Screening Test (ASSIST) [96], a measure of dependence on cannabis, cocaine, and other drugs over lifetime and past 3-months, with high levels of internal consistency, construct, concurrent and discriminant validity [97] and takes 15 minutes to complete]; and adverse childhood experiences [Adverse Childhood Experiences (ACE) [98] Questionnaire, consisting of 10 questions, with extensive reliability and validity data and takes less than 3 minutes to complete]. To get at patient strengths in the face of toxic stress, the Brief Resilience Scale (BRS) [99] will also be administered, a 6-item tool that with internal consistency ranging from .80–.91 and takes 3 minutes to complete. All questionnaire data will be collected at pre-assessments, post-assessments and at 3 months post-assessment follow-up by agency health and social service providers /ATTACH^TM^ interventionists, who will be trained and supervised to do so on REDCap (www.project-readcap.org) as part of the ATTACH^TM^ training program. Please see Additional file 3: Appendix 3 for Dried Blood Samples (DBS) Protocol for DBS collection.

### Data management {19}

Partner agencies will collect the data on REDCap provided by the University of Calgary (https://redcap.ucalgary.ca/) via iPads with REDCap software already installed. Agency health and social service providers /ATTACH^TM^ interventionists will be trained and supervised to employ the iPads and REDCap (www.project-redcap.org) as part of the ATTACH^TM^ training program. Partner agency health and social service providers /ATTACH^TM^ interventionists will be provided with a login information access the pre-, post-, and delayed post-assessment questionnaires. After logging in, they will ask the participant to fill out the questionnaires, which will only request de-identified data (except for required linkage to consent, filed separately); data will be automatically shared to the REDCap website after completion. Any data sharing or communication from the partner agencies will be done via the University of Calgary domain specific email account. Digital video data will be saved on the iPads and uploaded to secure Box on the cloud (https://www.box.com/en-ca/capture). The staff at the local agencies will be trained/ instructed to delete any digital data from the iPads. Digital copies of transcripts and audio-files will be kept in a secure network location administered by the University of Calgary’s IT services and accessible only to the research team.

All the information contained in our analyses and summaries will be anonymous and based on group data. Any report published as a result of this study will not identify the participants by name, address, or any other personal information. Furthermore, all research team members are aware of the importance of maintaining participant anonymity and are required to sign a confidentiality agreement.

### Statistical methods {20a, 20b and 20c}

We will analyze the demographic characteristics of the sample with measures of central tendency and frequencies as appropriate. For all analyses, alpha will be set a priori at .05 (two-tailed).

#### Objective 1: ATTACH^TM^ impacts

For (1a), Evaluate ATTACH^TM^ impacts on parent-child relationship quality (primary outcome), parental RF, and child development and (1d) evaluate long-term impact of ATTACH^TM^ (3 months delayed post-assessment), we will employ repeated measures analysis of variance (ANOVA), paired-t-tests, and chi-square tests to examine outcomes between pre-assessments, post-assessments and at 3 months post-assessment follow-up. For (1b) Determine whether ATTACH^TM^ is equally effective across patient populations (and for whom it works best/worst), we will examine differences among sub-groups derived from known covariates through use of independent samples *t*-tests (two groups, e.g., child sex), ANOVAs (more than 2 groups, e.g., race/ethnicity), repeated measures analysis of covariance (with identified covariate) and linear regression models (continuous covariate, e.g., age, years of education). For (1c) evaluate whether ATTACH^TM^ impacts immune biomarkers indicative of inflammatory disease risk, (i.e., gene expression, DNA methylation).

#### Objective 2: ATTACH^TM^ implementation feasibility

Analysis will occur in two parts. First, interviews will be transcribed and coded and examined for key learnings that may offer guidance to implementation in agencies. These learnings will be shared with the Executive Team, PEC, and CEC and discussed so that appropriate adjustments may be made to facilitate ATTACH^TM^ normalization in agencies. Second, all transcripts will be coded using the stages of thematic analysis including familiarization, coding, theme development, and data reporting [100]. Theme and sub-theme development will be deductive, using a priori codes dictated by interview questions to explain factors that promote or inhibit interventions from being embedded in agency practice. Two trainees will code the data, supervised by Dr. Letourneau, who is experienced in qualitative data analysis. Data will be managed with QSR International Nvivo12 [101]. Once themes and sub-themes are finalized, findings summarized in a draft report and shared with key informants as a validation check [101].

## Methods: monitoring

### Data monitoring {21a, 21b}

There is no data monitoring committee nor interim analysis since this is a social intervention, not the drug or pharmaceutical trial.

### Harms {22}

Any incidents (e.g., mental health crisis) observed by the investigators or facilitators will be recorded and managed as needed (e.g., with appropriate comfort measures and mental health referral). If the study staff interacting with these families observes any child abuse, they will report it to the Law Enforcement Authorities or act upon to intervene. The blood sample collection involves the potential for minor risk of discomfort, redness and swelling, and a rare risk of infection and fainting. However, these risks are small when blood samples are taken by trained staff, using standard blood sample collection procedures, and care will be taken to avoid these risks (see Additional file 3: Appendix 3). Moreover, due to the COVID-19 pandemic additional risks are associated with in-person participation.

### Auditing {23}

There is no data auditing since this is a social intervention, not the drug or pharmaceutical trial.

## Ethics and dissemination

### Research ethics approval and consent or assent {24 and 26a}

Ethics approval has been obtained from the Conjoint Health Research Ethics Board (CHREB; Ethics ID: REB20–0903) of the University of Calgary, and all participants will undergo a process of informed consent. The University of Calgary is the lead agency conducting the study and partner agency research sites rely on CHREB’s approval as part of their agency ethics protocols. All funding and research guidance flows from the University of Calgary and partner agencies will not have access to the study data, nor will they be involved in data analysis or data storage. The participants will be asked to provide informed consent. We have created different consent forms for the individual interviews and intervention participation to clearly indicate to patients what they are consenting to participate in (see Additional file 1: Appendix 1 and Additional file 2: Appendix 2).

The voluntary nature of the study will be reinforced verbally throughout the consent process and, indeed, throughout the course of the participant’s involvement in the study. They may choose not to answer some questions asked or to withdraw from the study at any time without affecting their health care or participation in partner agencies’ services. If they choose to no longer participate (at any time including once data analysis has begun), we will retain their data for attrition analyses, unless asked explicitly to remove data from the study, in which case their data collected to date will be destroyed. The interested agencies will approach the potential ATTACH^TM^ participants who are already accessing services at the agencies. Staff at the participating agencies will ensure to avoid any coercion by letting the potentially interested families know that their participation is completely voluntary, and that they can withdraw any time. It will not affect their access to any other services at the agencies if they decide to not participate in the study. To protect the rights of participants, this study will meet the ethical standards set out in the Conjoint Health Research Ethics Board Guidelines refs. A process of informed consent will be implemented, with verbal consent being secured at each stage of the process. In addition to the verbal consent secured during the recruitment and screening process, participants will be provided with a written consent form and will be required to return a signed copy of the written consent form.

We also received ethical approval for an adaptive honorarium schedule of gift cards that provides increased compensation commensurate with increased parent burden. This schedule emerged from numerous collaborative conversations with parents and agency health and social service administrators and providers. The study participants will receive $280 honorarium in total to appreciate them for their time. Additional $100 ($50 × 2 per sampling) will be offered to the families for dried blood sampling collection in the first 80 families in Calgary agencies. Participating agencies will cover the cost for parking and travel, as their typical practice.

### Protocol amendments {25}

We have not made any amendments to the protocol.

### Consent or assent {26b}

There are no additional consent provisions for collection and use of participant data and biological specimens in ancillary studies.

### Confidentiality {27}

To protect confidentiality and anonymity of findings, all data will be held in confidence, numerically coded with identifying information removed, and stored on a secure network drive. No personal email addresses or accounts will be used for communication purposes or for data sharing. The staff will ensure that the participant signs the consent form to understand the privacy and confidentiality nature of the study in the beginning. Additional steps may include reiterating the privacy and confidentiality nature of the study before digital video-recording taping.

Parents’ demographic information including name and age will be collected by the partner agencies. With the exception of the consent form (stored separately), only de-identified data will be shared with the ATTACH^TM^ team. The demographic information will be used to describe our sample in future publications. Interactions between the parents and children will be digitally video recorded to assess parent child interaction quality. These assessments are age specific. Any video digital data will be password protected or encrypted. The researchers will not have access to identifying information as participants will only be identified with an ID number. Any identifying information will be removed from the beginning and replaced with an ID number for analysis. Only the research team will have access to questionnaire response data. All information provided by participants will be kept confidential, except when it needs to be reported as required by law (when participants express a desire to do harm to themselves or others). No participant will be identified in any publications or presentations that come from this research. The findings of this study will be reported as aggregate data at health conferences and in journal publications; however, any information that could identify the participants will not be included.

### Declaration of interests {28}

The authors declare that they have no competing interests.

### Access to data {29}

The datasets used and/or analyzed during this study can be made available by the corresponding author upon request and in agreement with the research collaboration and data transfer guidelines of the University of Calgary and the ATTACH^TM^ researchers.

### Dissemination policy {31a, 31b, 31c}

The project will continue extensive iKT engagement with partners throughout all aspects of the project, resulting in various KT products including traditional peer-reviewed presentations in conferences and papers as well as more innovative engagement opportunities including in-services, press releases, and opinion-editorials. End-of-grant KT will include a similar set of KT products. Engagement with various SPOR-funded networks, will offer additional opportunities for meaningful KT. Specifically, iKT and end-of-grant dissemination is/will be planned in partnership with the Alberta SPOR Unit (AbSPORU), the Alberta Primary and Integrated Health Care Innovation Network (AB PIHCIN). Connection with the AB PIHCIN and their Southern and Northern practice-based research networks and partnering PIHCI Networks in SK and MB will specifically enable KT to primary health and social service providers including physicians and nurse practitioners. Our partnership with Alberta Health Services will also promote KT to knowledge users including public health nurses and other health and social service providers, and administrators/policy influencers.

## Discussion

Interventions that focus on promoting parental RF have great potential to improve outcomes for children exposed to toxic stressors [11, 102]. Our proposed work is guided by a strong foundation from the completed pilot work that employed the IDEAS Framework^TM^ [5, 18, 103]. Our research questions are original and employ novel methodology (EIH and NPT) to test ATTACH^TM^. Our own and others’ research have shown that RF-focused parenting interventions like ATTACH^TM^ have the potential to improve parent-child relationships, and child development and health in families affected by toxic stress. The proposed project will test whether ATTACH^TM^ has additional benefits for immune function, potentially the first prospective examination of the impact of a parenting intervention on immune function in the preschool period. We will employ NPT [86] to help “normalize” the delivery of ATTACH^TM^, to close the gap between knowledge creation and knowledge implementation of effective interventions in the community. Engaging parents, community agency health and social service providers and administrators in collaboration with researchers offers the opportunity to evaluate novel impacts (immune & epigenetic) and promote the integration of ATTACH^TM^ into routine care provided at community agencies serving families at high psychosocial risk. We have proposed a feasible design and deliverables, based on past and planned engagement to answer the research questions.

**Fig. 1:**
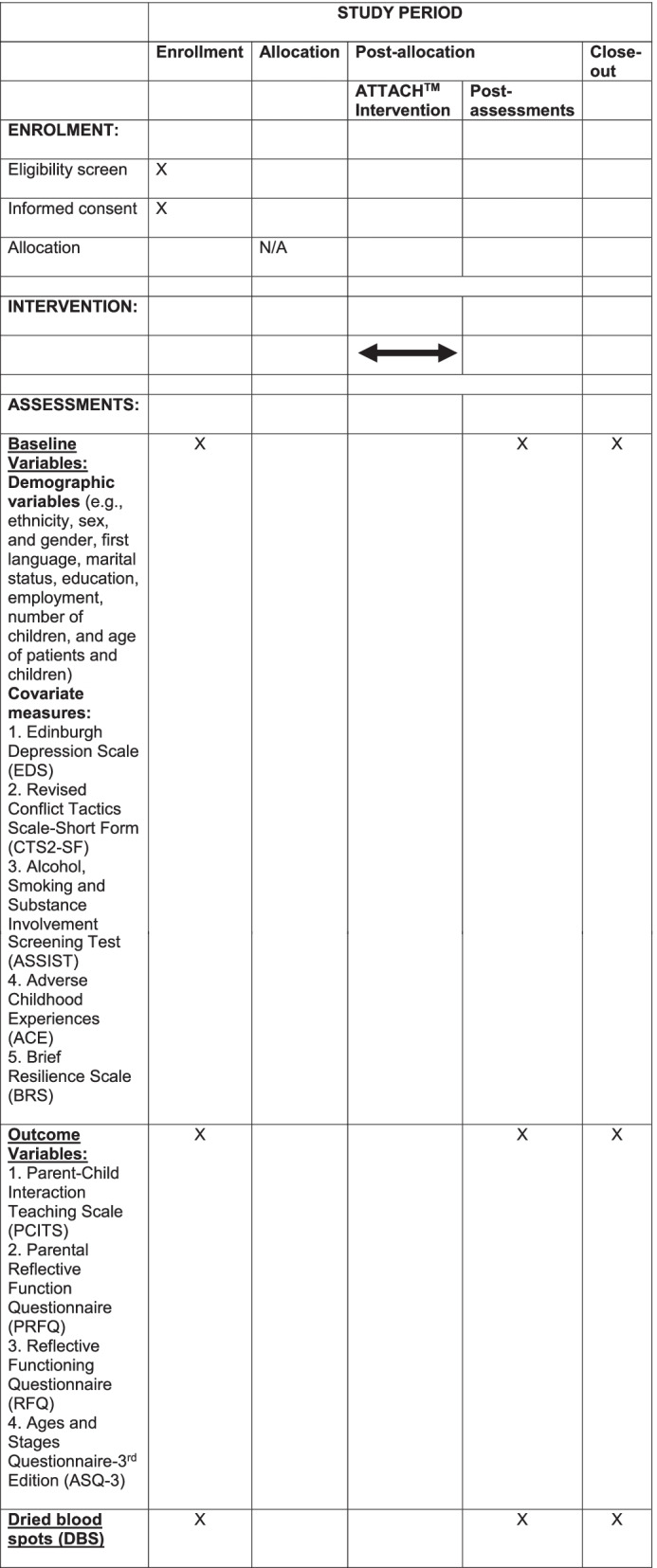
The Schedule of Enrolment, Intervention, and Assessment

For many children, toxic stress imposes a huge burden on their cognitive development (e.g., communication, problem-solving social skills) and physical health over the lifespan [104, 105], with costs to society in terms of lower school achievement and higher rates of chronic disease [85]. According to the National Academies of Science [17], the roadmap to health equity for children at risk due to early adversity and toxic stressors includes: (1) effective interventions that begin early, support caregivers, and maximize the potential of early care and education to support health outcomes—addressed by Objective 1; (2) reforming health care system services to promote healthy development—addressed by Objective 2; and (3) implementing initiatives to support children, families, other caregivers and communities of trauma-informed care—addressed by Objective 2. We stand to make an impact on parent experiences and outcomes via our parent partners, PEC, and CEC by implementing best practice approaches to patient engagement that will maximize the potential for ATTACH^TM^ to be feasible, acceptable, and effective for parents at risk in community settings (Fig. 1).

## Protocol version

Version 1.

## Status of trial

Recruitment in progress; start date of recruitment: November 16, 2021.

## Supplementary Information

**Additional file 1 Appendix 1.** Consent Form for Quantitative Component (Objective 1) {32}.

**Additional file 2 Appendix 2.** Consent Form for Qualitative Component (Objective 2) {32}.

**Additional file 3 Appendix 3.** Dried Blood Samples (DBS) Protocol {33}.

## Data Availability

The datasets used and/or analyzed during this study can be made available by the corresponding author upon request and in agreement with the research collaboration and data transfer guidelines of the University of Calgary and the ATTACH^TM^ researchers. Materials including all relevant raw data, will be freely available to any scientist wishing to use them for non-commercial purposes, without breaching participant confidentiality.

## Footnotes

1 We are adhering to the CIHR/SPOR Patient Engagement Guidelines, which defines a patient as an “individual with personal experience; and informal caregiver, including family member or friend”
