# Supplementary material for: Study protocol for Attachment & Child Health (ATTACHTM) program: promoting vulnerable Children’s health at scale
Source: BMC Pediatr. 2022 Aug 19;22:491. doi: 10.1186/s12887-022-03439-3 (PMC9388995; doi:10.1186/s12887-022-03439-3)
Supplement: Supplementary file 2 — Additional file 2 Appendix 2. Consent Form for Qualitative Component (Objective 2) {32}. [file 12887_2022_3439_MOESM2_ESM.docx]

**Appendix 2. Consent Form for Qualitative Component (Objective 2) {32}**

**CONSENT FORM**

**TITLE: Attachment and Child Health (ATTACH^TM^): Promoting Vulnerable Children’s Health at Scale**

**FUNDER:** The Canadian Institutes of Health Research (CIHR) Strategy for Patient-Oriented Research (SPOR)

**This consent form is only part of the process of informed consent. It should give you the basic idea of what the research is about and what your participation will involve. If you would like more detail about something mentioned here, or information not included here, please ask. Take the time to read this carefully and to understand any accompanying information.**

**BACKGROUND**

Attachment refers to the kind of relationship a baby or child has with their parent. For parents suffering from stress like depression, addictions, and/or family violence, it may be hard to care for their babies/children the way they would like to if they were not stressed. This can affect the kind of attachment the baby/child has with their parent. “Secure” or “good” attachment is shown when a baby/child can rely on their parent for safety and comfort.

Our program helps babies and children become secure by helping their parents with their reflective function. Reflective function is the ability to recognize and identify feelings and thoughts in oneself and their child. Learning to enhance this skill of reflective functioning can influence parents’ actions toward their child.

The ATTACH^TM^ parenting program is designed to help parents with their reflective function by practicing this skill in an effort to promote secure attachment in their babies. This, we hope, will improve child health and development. We invite you to take part in this program and the program evaluation as part of a research study.

**WHAT DOES REFLECTIVE FUNCTION DO?**

When parents have the ability to recognize their own feelings/thoughts and their child’s feelings/thoughts, this helps parents:

- react positively
- predict what their baby/child needs
- change how the parent responds
- to help their baby/child feel safe and secure

**WHAT IS THE PURPOSE OF THE STUDY?**

The purpose of this study is to assess your perceptions and experiences of the Attachment and Child Health (ATTACH™) parenting program. We will ask you questions about: (1) the benefits, barriers, and challenges of implementation and (3) the utility of ATTACH™ e-learning platform. The ATTACH^TM^ parenting program is a trademarked program; the program developers may use it for incorporated purposes.

**WHAT WOULD I HAVE TO DO?**

This study is conducted in collaboration with our partner community agencies. You have been asked to provide general information about yourself and take part in a one-on-one interview session by telephone, Zoom meeting, or in person. We will seek your point of view about the ATTACH™ intervention benefits, facilitators, barriers, and challenges to implementation by community agencies. We will also seek your feedback on program materials, such as the fidelity checklist (to examine the accuracy of implementation of ATTACH^TM^) and e-learning training, as appropriate. The interview will take approximately 60 minutes. We will also ask for your permission to audio and/ or video record the interview. The interviews may be done via Zoom. Zoom data will be kept secure by encryption and password protection.

**WHAT ARE THE RISKS OF PARTICIPATION?**

Risks involved with this study are minimal. You may become distressed while you are in the study. If this happens, the agency staff will help you identify any extra help you need. If at any point during the study you feel you need some help with your mental health, please call your family doctor, or your local crisis/ distress Line or mental health services. Also, risks may be associated with breach of privacy or disclosure of personal information which will be minimized by recording data locally, password protection, and using institutional account.

- Due to the COVID-19 pandemic, additional risks are associated with in-person participation. Please let the ATTACH^TM^ facilitator know if you have any questions or concerns. To ensure the risks are minimal for you, the following measures will be done:

1) The day before or the morning of your appointment, a member of the ATTACH^TM^ team will call you and do the Alberta Health Services self-assessment tool for COVID-19. The self-assessment tool can be found online using this link (<https://myhealth.alberta.ca/journey/covid-19/Pages/COVIDSelf-Assessment.aspx>).

2) On your appointment day, the following will be done:

- Use of Personal Protective Equipment (PPE) is required for both the ATTACH^TM^ facilitator and research participants (e.g., masks, gloves). Clear masks are required for the study as we observe facial cues of participants. The clear masks are FDA approved (https://www.theclearmask.com/) and will be used by both the ATTACH^TM^ facilitator, you, and your child. Children under 2 years of age will not be required to wear a mask. We will provide you with the clear masks. Further, your ATTACH™ facilitator, who is affiliated with the agency where you learned about ATTACH™ will follow their own COVID-19 agency health and safety standards.
- Use of hand sanitizer for both you and the ATTACH^TM^ facilitator, will be provided by the study team.
- We will ask you to provide toys for your baby to use for the program play sessions.
- Physical distancing measures of 2m will be followed during the sessions.
- Surfaces and multi-use equipment will be sanitized by the ATTACH^TM^ facilitator between uses.

Incremental research related risks of exposure may include:

- increased time within a health care facility,
- increased exposure to other people (e.g., patients, participants, or people).

Where participants are visiting the agencies facilities for a research interaction only, research-related risks may include:

- risks associated with travel (e.g., public transit),
- time within a health care facility, and.
- exposure to other people.

**ARE THERE BENEFITS TO PARTICIPATING?**

If you agree to participate in this study, there may or may not be a direct benefit to you. The information we gather may help us to evaluate the ATTACH^TM^ program effectiveness, which will help to promote healthy development of children. This information is valuable because it may contribute to our understanding of how to improve the health and wellbeing of mothers and their children.

**DO WE HAVE TO PARTICIPATE?**

No. You are under no obligation to participate. Participation may be terminated at any time. You may withdraw by telephone, email, or in person. If participating could potentially pose a health or safety risk, then the researcher may withdraw you from the study. You will also be informed if any new information becomes available that may affect participation. If you withdraw, we will keep your data collected to that point unless you ask us to withdraw it. Data cannot be withdrawn once made anonymous for analysis. If you would like to withdraw, please contact the Project Manager, Dr. Martha Hart (403) 681-3592.

**WILL I BE PAID FOR PARTICIPATING, OR DO I HAVE TO PAY FOR ANYTHING?**

Participating agencies will cover the cost for parking and travel, as their typical practice.

**WILL RECORDS BE KEPT PRIVATE?**

Yes. All data that you contribute to this study will be kept completely confidential. Data will be entered into the computer with a subject number. Original audio and/ or video files will be saved on encypted hard drives that will be stored in locked file cabinets in Owerko Centre, Child Development Centre at the University of Calgary. Any reports based on your information will be combined with data from other participants so that it will not be possible to identify any individual. Under Alberta legislation, all citizens, including agency's staff, must contact the proper authorities if a child is in danger and/or if there is a risk of self-harm or of harm to others. All data transferred from agencies will be de-identified, and encrypted.

**IS THERE ANY COMPENSATION FOR A RESEARCH-RELATED INJURY?**

No. No compensation will be provided to you by the University of Calgary, ATTACH^TM^, or the participating agencies, if you should suffer any research-related injury. However, you still have all your legal rights. Nothing said in the consent form alters your right to seek damages.

**SIGNATURES**

In signing this form, you affirm that you have understood the information regarding your participation in this study to your satisfaction. Signing this form does not waive legal rights nor does it release the investigators or involved institutions from their legal and professional responsibilities.

As stated previously, participation can be terminated at any time. If you have any questions related to this research, please contact Dr. Nicole Letourneau (403) 210-3833 or Dr. Martha Hart (403) 681-3592. If you have any questions concerning your rights as a possible participant in this research, or research in general, please contact the Chair of the Conjoint Health Research Ethics Board, University of Calgary at (403) 220-7990.

_______________________________ ________________________________

Participant’s Name Signature and Date

_______________________________ ________________________________

Investigator’s Name Signature and Date

_____________________________ ________________________________

Witness’ Name Signature and Date

*The University of Calgary Conjoint Health Research Ethics Board has approved this research study.*

*A signed copy of this consent form has been given to you to keep for your records and reference.*

*Public Information about this Study:
ClinicalTrials.gov is a website that provides information about federally and privately supported clinical trials. A description of this clinical trial will be available on http://www.ClinicalTrials.gov. This website will not include information that can identify you. At most, the website will include a summary of the results. You can search this website at any time.*
