# Supplementary material for: Study protocol for Attachment & Child Health (ATTACHTM) program: promoting vulnerable Children’s health at scale
Source: BMC Pediatr. 2022 Aug 19;22:491. doi: 10.1186/s12887-022-03439-3 (PMC9388995; doi:10.1186/s12887-022-03439-3)
Supplement: Supplementary file 3 — Additional file 3 Appendix 3. Dried Blood Samples (DBS) Protocol {33}. [file 12887_2022_3439_MOESM3_ESM.docx]

**Appendix 3. Dried Blood Samples (DBS) Protocol {33}**

Location:

Community or partner agencies

Supplies:

- x2 DBS Card (Whatman 903 Protein Saver Cards) – write participant ID and date on card prior to blood sample collection
- Alcohol swabs
- Sterile gauze
- Disposable finger lancet (supplier TBD)
- Band aids
- Gloves
- Sharps container, biohazard garbage bag

Additional supplies (to help with sample collection):

- Electric blanket (to help promote circulation)
- EMLA cream (to be tested before hand to see if it affects capillary blood flow)
- Wrist buzzer (distraction toy placed on child’s wrist before blood collection)

Child finger prick (Note: need 2-4 spots, depending on how large the spots are)

- If the participant choses to use a topical anesthetic, apply to the finger five minutes before the blood collection protocol
- Ask participant to rub their hands to warm them up (increase blood flow) and swing their hands rapidly downward
- *For children: Provide the child with “wrist buzzer” distraction and/or start a video/game on the iPad. Mother and a research assistant will provide additional distraction and comfort.
- Set up for the finger prick: x3 alcohol swabs, x2 gauze, lancet, DBS card
- Wash hands and put on gloves
- Ask the participant to put their hand in a “gun” position
- Wipe the participant’s middle finger with alcohol (have them turn their palm up and especially wipe the innermost side of the finger)
- Prick the middle finger (just off the center of the tip of the finger, close to the pad) with a lancet
- Wipe away the first drop of blood with a gauze pad and apply drops to filter paper. Do not touch the participant’s skin to the filter paper – rather, allow the blood to well and wick the blood onto the filter paper (if the skin touches the paper, the resulting spot will be uneven); rotate the card is it is in-line with the participant’s finger and fill 4-5 spots on the DBS card, depending on blood flow
- Note: Frequently wipe away blood from the puncture site (avoid clotting)
- Note: If blood flow is not satisfactory, try squeezing the finger slightly; DO NOT milk the finger (excessive squeezing) as this will dilute the sample; if blood flow is still insufficient, end the finger prick and try again on another finger (e.g., ring finger)
- When the DBS cards are completed, wrap gauze around the finger and ask the participant to apply pressure
- Discard materials contaminated with blood in the biohazard garbage, dispose of the lancet in sharps
- Apply a band aid to the participant’s finger

Mother finger prick (Note: need 2-4 spots, depending on how large the spots are)

- If the participant choses to use a topical anesthetic, apply to the finger five minutes before the blood collection protocol
- Ask participant to rub their hands to warm them up (increase blood flow) and swing their hands rapidly downward
- Set up for the finger prick: x3 alcohol swabs, x2 gauze, lancet, DBS card
- Wash hands and put on gloves
- Ask the participant to put their hand in a “gun” position
- Wipe the participant’s middle finger with alcohol (have them turn their palm up and especially wipe the innermost side of the finger)
- Prick the middle finger (just off the center of the tip of the finger, close to the pad) with a lancet
- Wipe away the first drop of blood with a gauze pad and apply drops to filter paper. Do not touch the participant’s skin to the filter paper – rather, allow the blood to well and wick the blood onto the filter paper (if the skin touches the paper, the resulting spot will be uneven); rotate the card to ensure it is in-line with the participant’s finger and fill 4-5 spots on the DBS card, depending on blood flow
- Note: Frequently wipe away blood from the puncture site (avoid clotting)
- Note: If blood flow is not satisfactory, try squeezing the finger slightly; DO NOT milk the finger (excessive squeezing) as this will dilute the sample; if blood flow is still insufficient, end the finger prick and try again on another finger (e.g., ring finger)
- When the DBS cards are completed, wrap gauze around the finger and ask the participant to apply pressure
- Discard materials contaminated with blood in the biohazard garbage, dispose of the lancet in sharps
- Apply a band aid to the participant’s finger

Post-collection procedure:

- Leave the DBS card open in a separate wet-lab space and allow to air dry for at least four hours (or overnight)
- After the card is dried, close the card and store at -30C.
- Samples will be stored at the Owerko Centre until shipment to the UCLA Social Genomics Core Laboratory at the University of California, Los Angeles, to be assayed by Dr. Steve Cole, PhD.
